# Ability of hydrogen storage CeNi_5-*x*_Ga*_x_* and Mg_2_Ni alloys to hydrogenate acetylene

**DOI:** 10.1080/14686996.2019.1629836

**Published:** 2019-06-12

**Authors:** Ryota Tsukuda, Ryo Yamagishi, Satoshi Kameoka, Chikashi Nishimura, An-Pang Tsai

**Affiliations:** a Institute of Multidisciplinary Research for Advanced Materials, Tohoku University, Sendai, Japan; b Department of Materials Processing, Graduate School of Engineering, Tohoku University, Sendai, Japan; c Center for Green Research on Energy and Environmental Materials, National Institute for Materials Science, Tsukuba, Japan

**Keywords:** Intermetallic compound catalyst, hydrogen storage alloy, hydrogenation of acetylene, CeNi_5-*x*_Ga*_x_*, Mg_2_Ni, reactivity of hydride, 50 Energy Materials, 106 Metallic materials, 205 Catalyst / Photocatalyst / Photosynthesis, 206 Energy conversion / transport / storage / recovery, 212 Surface and interfaces, 504 X-ray / Neutron diffraction and scattering

## Abstract

Hydrogen storage properties and reactivity for hydrogenation of acetylene in a series of CeNi_5-*x*_Ga*_x_* (*x* = 0, 0.5, 0.75, 1, 1.25, 1.5) alloys and Mg_2_Ni were determined and compared. The structure of CeNi_5_ (CaCu_5_ type) was maintained up to CeNi_3.5_Ga_1.5_ when Ni was replaced by Ga. The replacement facilitated hydrogenation absorption by creating larger interstitial spaces through expansion of the lattice, allowing CeNi_4.25_Ga_0.75_ to absorb the greatest proportion of hydrogen atoms among the alloys under the same conditions. The results showed that the absorbed hydrogen in CeNi_3.75_Ga_1.25_ improved reactivity. In contrast, Mg_2_Ni formed a hydride upon hydrogenation of acetylene and thus possessed much lower activity. The difference of the activity of absorbed hydrogen between CeNi_5-*x*_Ga*_x_* and Mg_2_Ni was confirmed from transient response tests under reaction gases alternately containing He and H_2_.

## Graphical abstract

**Figure:**
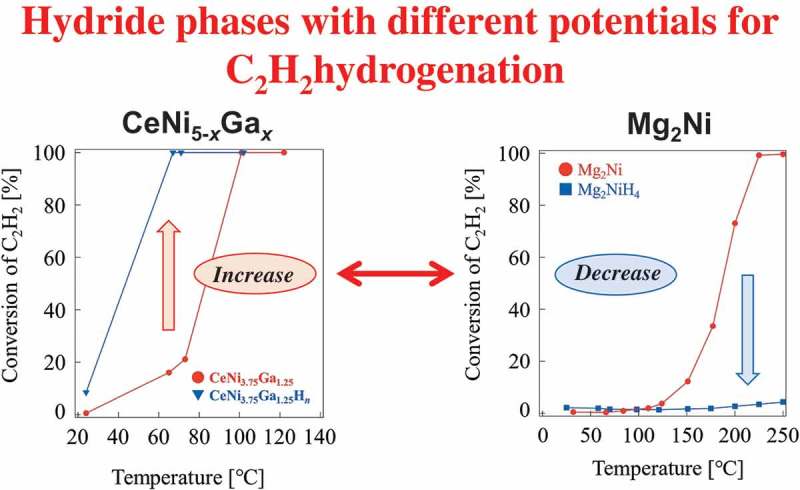

## 1. Introduction

Hydrogen is an ideal candidate for alternative energy resources because of its portability and low environmental impact. Hydrogen storage is the most important factor in the development of applications. Therefore, many hydrogen storage alloys have been investigated to improve the capacity, kinetics and cyclic behavior [1].

The RENi_5_ (RE = rare earth) intermetallic compounds (IMCs), e.g. LaNi_5_, are hydrogen storage alloys that are used in practical applications, such as hydride batteries [2,3]. The advantages of RENi_5_ IMCs are their high hydrogen absorption/desorption kinetics. Substitution of group 13 elements for Ni may change the hydrogen capacity, equilibrium plateau pressures, and stability of their hydrides. Many intermetallic RENi_5_ in which Ni was replaced with Al [4–12], Ga [13–18], or In [19], have been examined for their potential to improve hydrogen absorption properties. In these IMCs, the equilibrium plateau pressures generally decrease as Ni substitution increases. Therefore, these IMCs may be able to be tuned to absorb hydrogen at an arbitrary temperature and pressure by changing additional elements. Physical properties, such as magnetic susceptibility, of CeNi_5-*x*_Ga*_x_* alloys have been determined [20,21], although only the hydrogen storage properties of CeNi_4_Ga were reported [18]. Thus, one goal of the present study was to investigate the hydrogen absorption properties of a series of CeNi_5-*x*_Ga*_x_* (*x* = 0, 0.5, 0.75, 1, 1.25, 1.5) alloys.

Dissociative oxygen atoms or hydrogen atoms at the subsurface are active in chemical reactions [22,23]. A number of studies have reported that absorbed hydrogen is essential for hydrocarbon hydrogenation, with Pd containing absorbed hydrogen showing high reactivity [24–31]. Since the state of absorbed hydrogen is similar to a dissociative hydrogen atom, hydrogen storage alloys have been used as catalysts for hydrogenation of C_2_H_4_ and CO_2_ [32–36]. Using a hydrogen storage alloy as a catalyst is a new approach to catalysis, however, few studies about hydrogen storage alloys and the selectivity of products from catalysis for hydrogenation of alkynes have been reported [4]. The present study describes the development of a series of CeNi_5-*x*_Ga*_x_* alloys as hydrogen storage alloy catalysts and their hydrogen absorption properties and catalytic reactivity upon substitution with Ga. In addition to CeNi_5-*x*_Ga*_x_* alloys, Mg_2_Ni IMC was also investigated for comparison because it is a well-studied hydrogen storage alloy. Previous studies have indicated that the hydrogen absorption/desorption kinetics for Mg_2_Ni are lower than those for RENi_5_ IMCs. Magnesium-based alloys have a high potential for hydrogen storage in terms of hydrogen-absorption capacity [37]. In many aspects, the interactions of H-Mg_2_Ni are different from those of H-RENi_5_, and so investigating the activity of hydrogen atoms in a given catalytic reaction is interesting. Therefore, another goal of this study was to investigate the catalytic properties of hydrogenation of C_2_H_2_ over CeNi_5-*x*_Ga*_x_* alloys and Mg_2_Ni.

## 2. Experimental procedures

### 2.1. Material preparation

Pure Ce, Ni, and Ga were mixed in appropriate ratios to prepare a series of CeNi_5-*x*_Ga*_x_* alloys (*x*= 0, 0.5, 0.75, 1, 1.25, 1.5) in an arc-melting furnace under an Ar atmosphere. Element purities were: Ce 99.9%, Ni 99.99%, Ga 99.9999%. The as-cast alloys were annealed at 800°C for 72 h under an Ar atmosphere for homogenization. Afterward, annealed samples were sieved under 20 μm. Structural analysis and phase identification were performed using X-ray powder diffractometry (Rigaku, Ultima IV, Japan) with Cu-Kα radiation. The X-ray diffraction (XRD) data were analyzed using the Rietveld method to calculate the lattice parameters of IMCs and to determine the substitution site of Ga. The FULLPROF program was used for analyzing the data obtained [38]. For calculation of X-ray diffraction, the space group P6/mmm (No. 191) of the hexagonal crystal system was used.

The Mg (99.9%) and Ni (99.99%) were placed into a Tammann tube at specified proportions and Mg_2_Ni was prepared in an induction-melting furnace under an Ar atmosphere. The as-cast alloys were annealed at 550°C for 72 h under an Ar atmosphere. The Mg_2_Ni was passed through a sieve to achieve a particle size in the range of 38 − 63 μm. The hydride phase of Mg_2_NiH_4_ was synthesized using a standard Sieverts apparatus. The Mg_2_Ni was placed into a stainless vessel. Hydrogen at a pressure up to 4 MPa was introduced into the Mg_2_Ni at 350°C followed by evacuation of the vessel. This absorption/desorption process was repeated five times. The vessel was cooled slowly after introducing a hydrogen pressure of 4 MPa. The XRD analyses of CeNi_5-*x*_Ga*_x_* alloys were performed using the same equipment as for the alloys mentioned above. The SEM was employed to determine the morphology of powder specimens using a Hitachi SU 8000 instrument.

### 2.2. Measurement of hydrogen storage properties

Hydrogen storage properties of CeNi_5-*x*_Ga*_x_* alloys (*x* = 0, 0.5, 0.75, 1, 1.25, 1.5) were investigated by the volumetric method using a standard Sieverts apparatus (Suzuki Shokan, Japan). The samples, sieved to a size in the range of 25 − 90 μm, were placed in a stainless vessel. Initial activation procedures involved two processes: 1) hydrogen gas introduction into the vessel up to 3 MPa, followed by heating of the samples at 300°C for 1 h; 2) then, hydrogen absorption/desorption cycles up to 4 MPa were repeated several times at 75°C. After these pretreatments, pressure-composition-isotherm (PCI) measurements were obtained at 25°C, 50°C, and 75°C. Furthermore, *in situ* XRD analyses of CeNi_4_Ga and CeNi_3.75_Ga_1.25_ were performed (PANalytical, Empyrean, Netherlands). The powder samples were transferred into a silicon container and then held at room temperature for 30 min under vacuum before being heated at 300°C for 1 h under hydrogen pressures up to 1MPa, and finally cooled to room temperature. *In situ* XRD data were acquired during the desorption process under various hydrogen pressures at room temperature.

### 2.3. Catalytic test

Catalytic tests were conducted using CeNi_5_, CeNi_3.75_Ga_1.25_, and CeNi_3.5_Ga_1.5_ in the Ce-Ni-Ga system. Three samples were sieved to a size in the range of 25 − 90 μm. The standard Sieverts apparatus was used for pretreating the three samples, which involved: introduction of hydrogen pressure up to 3 MPa into the alloys at 300°C that was maintained for 1 h; after evacuating and cooling at 25°C, hydrogen absorption/desorption cycles up to 4 MPa were repeated several times for CeNi_5_ and CeNi_3.75_Ga_1.25_; the same absorption/desorption cycles were conducted for CeNi_3.5_Ga_1.5_ at 75°C. Afterward, the three samples were referred to as CeNi_5_, CeNi_3.75_Ga_1.25_H*_n_*, and CeNi_3.5_Ga_1.5_H*_n_*, respectively. The PCI measurements showed that the initial activations for CeNi_5-*x*_Ga*_x_* alloys were needed to achieve hydrogen pressure greater than 1 MPa, which allowed comparison of an alloy with and without absorbed hydrogen, depending on the condition of initial activation. Accordingly, in this study, CeNi_3.75_Ga_1.25_ was prepared without introducing high hydrogen pressure. The catalytic test was performed using a standard ﬁxed-bed ﬂow reactor. The four samples, CeNi_5_, CeNi_3.75_Ga_1.25_H*_n_*, CeNi_3.5_Ga_1.5_H*_n_*, and CeNi_3.75_Ga_1.25_, were each charged into a straight quartz tube. The CeNi_5_, CeNi_3.75_Ga_1.25_H*_n_*, and CeNi_3.5_Ga_1.5_H*_n_* contacted air once when they were moved to the standard ﬁxed-bed ﬂow reactor from the Sieverts apparatus. The samples were activated under a pure hydrogen flow of 0.1 MPa at 30 mL min^–1^ and heated at 300°C for 1 h. After that, the samples were held at 75°C for 1 h under flowing H_2_ and cooled to room temperature. The reaction gases passed over CeNi_5-*x*_Ga*_x_* alloys were 0.67%C_2_H_2_/6%He/93.33%H_2_ (*P*_H2_ = 0.093 MPa). Total flow rate and pressure were fixed to 30 mL min^–1^ and 0.1 MPa, respectively.

Similarly, Mg_2_Ni and Mg_2_NiH_4_, which has a desorption temperature higher than 250°C [39], were charged into straight quartz tubes. Initial activation of each alloy was conducted under a flow of pure H_2_ at 30 mL min^–1^ and 0.1 MPa, followed by maintaining Mg_2_Ni at 300°C for 1 h and Mg_2_NiH_4_ at 200°C for 1 h, under the same hydrogen flow rate. Then, the Mg_2_Ni and Mg_2_NiH_4_ were treated with 2%C_2_H_2_/18%He/80%H_2_ reaction gases (*P*_H2_ = 0.8 MPa). Total flow rate and pressure were 30 mL min^–1^ and 0.1 MPa, respectively. In addition, Mg_2_NiH_4_ was heated at 500°C under a H_2_ flow at 30 mL min^–1^ and kept for 2 h to release absorbed hydrogen. Afterward, the same catalyst test was conducted with the heat-treated Mg_2_NiH_4_.

The products were analyzed by in-line gas chromatography (GC; Shimadzu, GC-8 A, Japan) with a Shincarbon ST column (for C_2_H_2_, C_2_H_4_ and C_2_H_6_ analysis) with a He carrier gas. The conversion of C_2_H_2_ and the selectivity of C_2_H_4_ were defined as:

$$C_{C2H2}\left[\%\right]\;=\;\left({\left[C_{2}H_{2}\left(feed\right)\right]}_{mol}-\;{\left[C_{2}H_{2}\right]}_{mol}\right)/\text{\textbackslash{}break}\;\;\;\;\;\;\;\;\;{\left[C_{2}H_{2}\left(feed\right)\right]}_{mol}\times \;100$$

$$S_{C2H4}\left[\%\right]\;=\;{\left[C_{2}H_{4}\right]}_{mol}/\left({\left[C_{2}H_{4}\right]}_{mol}+\;{\left[C_{2}H_{6}\right]}_{mol}\right)\times \;100$$

where [C*_x_*H*_y_*]_mol_ represents experimental mol concentrations of C_2_H_2_, C_2_H_4_, and C_2_H_6_ in the outlet gases, and [C_2_H_2_(feed)]_mol_ represents mol concentration of C_2_H_2_ in the feed stream. Catalytic activity was monitored every 15 min.

Surface area of the powder catalysts (a_BET_) was estimated using the Brunauer-Emmett-Teller (BET) method with Kr adsorption.

### 2.4. Transient response test

Absorbed hydrogen in bulk alloys is consumed in the hydrogenation of C_2_H_2_ and catalytic reactions are thought to occur only with flowing C_2_H_2_ and He but without H_2_ in the reaction gas. Therefore, transient response tests were conducted by alternating C_2_H_2_+He and C_2_H_2_+H_2_ in order to clarify whether the absorbed hydrogen alone can drive the hydrogenation reaction in absence of H_2_ supply from the gas phase. The CeNi_5_, CeNi_3.75_Ga_1.25_H*_n_*, Mg_2_Ni, and Mg_2_NiH_4_ were chosen as samples for transient response tests. The initial activation process for each sample was the same as that for the catalytic test. The reaction gases 0.67%C_2_H_2_/6%He/93.33%H_2_ and 0.67%C_2_H_2_/99.33%He were switched alternately. Measurement temperature for the Ce-Ni-Ga system was 75°C and for the Mg-Ni system was 200°C. The definition of conversion rate was the same as that for the catalytic test.

## 3. Results and discussion

### 3.1. Crystal structure of CeNi_5-__x_Ga_x_ (x = 0, 0.5, 0.75, 1, 1.25, 1.5) alloys

**Figure 1.**
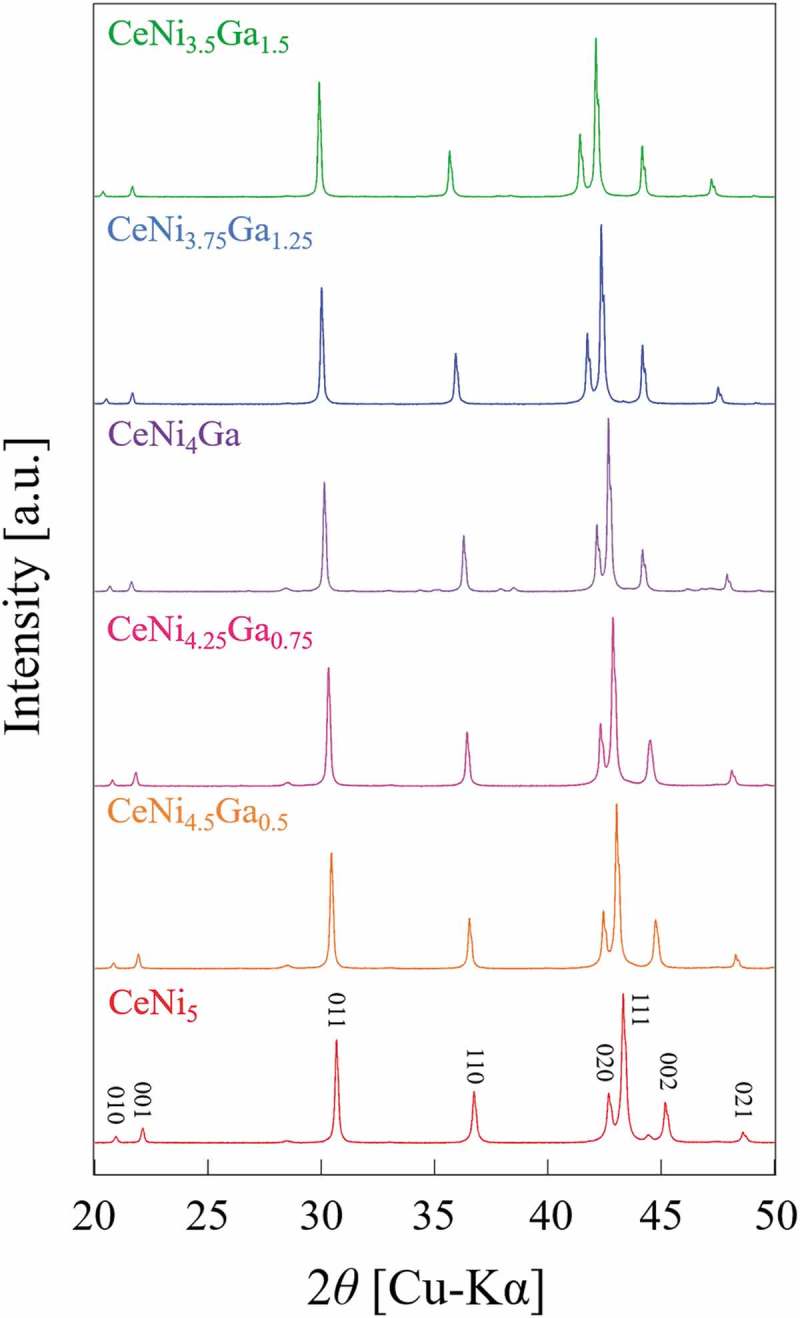
XRD patterns of CeNi_5-*x*_Ga*_x_* alloys.

**Figure 2.**
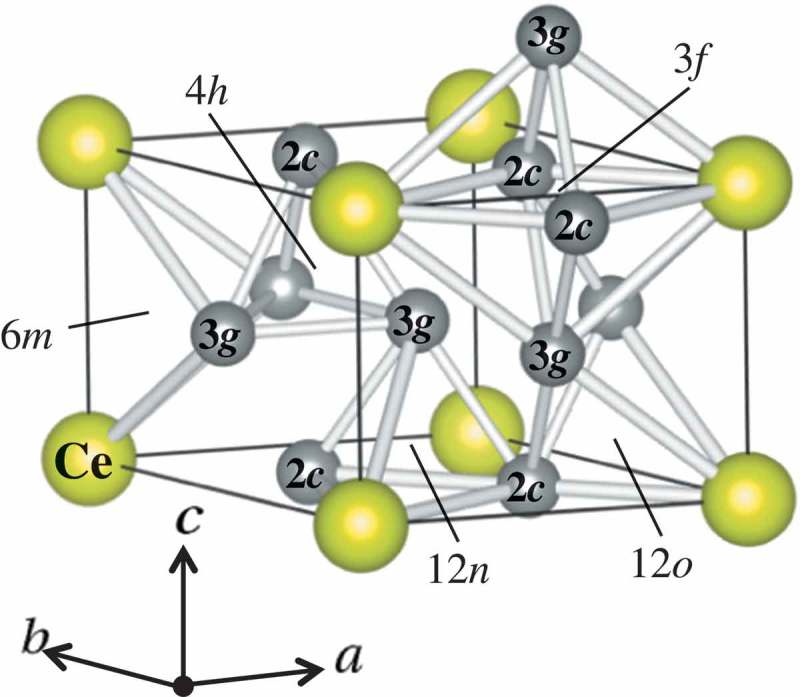
Schematic of the CaCu_5_ type structure and five interstitial sites (3*f*, 4*h*, 6*m*, 12*n*, and 12*o*) that hydrogen can occupy. Drawing by VESTA [40] and reproduced by permission from [4], Copyright Int. J. Hydrogen Energy, 2017.

**Figure 3.**
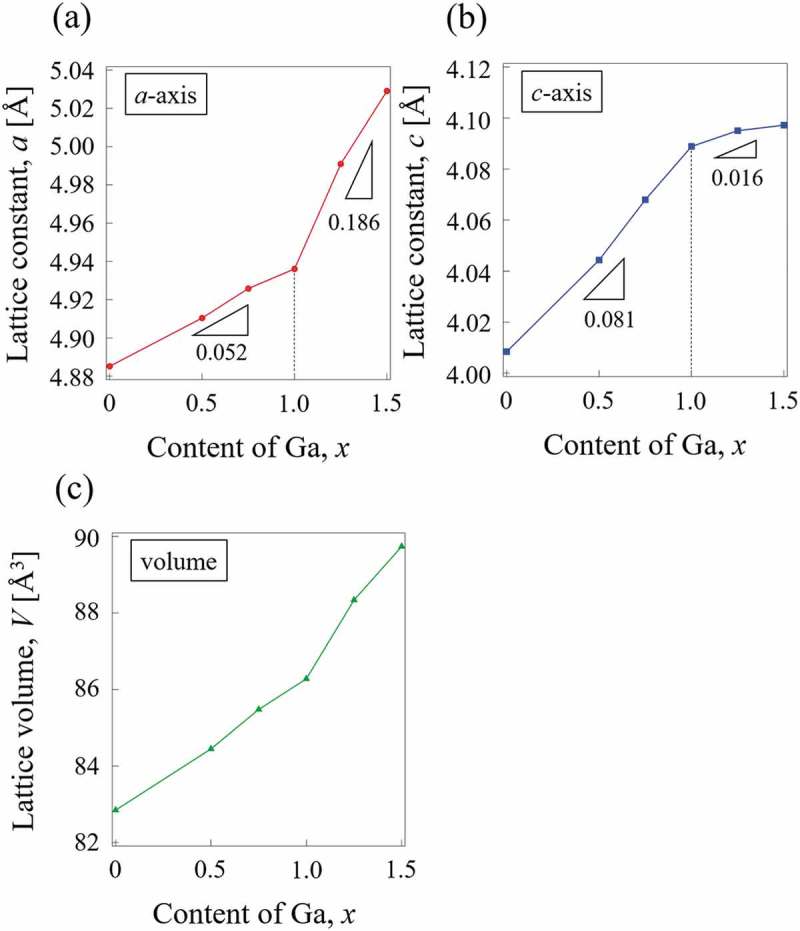
Variation in lattice parameters and unit cell volume: (a) *a*-axis, (b) *c*-axis, (c) unit cell volume.

The powder XRD patterns for CeNi_5-*x*_Ga*_x_* (*x* = 0, 0.5, 0.75, 1, 1.25, 1.5) alloys are shown in Figure 1. The results show that all alloys possess the same hexagonal structure (CaCu_5_-type) with a space group of P6/mmm. In the CeNi_5_ structure, Ce atoms occupy vertices site (1a site) and Ni atoms occupy two different atomic sites, one locates on the z = 0 plane (2c site) and the other locates on the z = 1/2 plane (3g site) as shown in Figure 2 [4,40]. Neutron diffraction shows that Ga preferentially substitutes strongly into the 3g sites of CeNi_4_Ga and CeNi_3.5_Ga_1.5_ [41]. Substitution preference appears to be governed by size factors; larger atoms such as Al and Ga preferentially occupy the 3g, while smaller atoms tend to substitute at the 2c site. In the present study, Rietveld analysis also indicates that all of the Ga atoms occupy 3g sites, which agrees with a previous report [41] and other RE-Ni-Ga systems [14–18]. The lattice parameters and unit cell volume of CeNi_5-*x*_Ga*_x_* alloys determined by Rietveld analysis are shown in Table 1 and Figure 3. Since the atomic radius of Ga is larger than that of Ni, the unit cell volume increases almost linearly with increasing Ga content. However, the increment of replacement amount of Ga (*x*) is different for the *a*-axis and *c*-axis. A refraction point occurs at *x* = 1 in both Figure 3(a,b): the slope of the lattice expansion increases at *x* > 1 for the a-axis but decreases in case of the *c*-axis. A similar trend is observed for CeNi_5-*x*_Al*_x_* alloys [4]. The substitution sites changed from the intermediate layer (3g sites) to the basal layer (2c sites) at *x* = 1 in LaNi_5-x_Ga_x_ [13]. However, Ga is supposed to occupy only 3g sites, which is observed not only in this study but also in another report [41]. This could be realized in terms of the atomic position of the CeNi_5_ structure, as shown in Figure 2. Three equivalent Ni (3g) atoms are located on the z = 1/2 layer, and Ga atoms are likely to substitute at the Ni position without forming any adjacent pair of Ga-Ga. In this manner, shown in Figure 2, Ga would selectively substitute a specific Ni 3g site at the body center of the unit cell. These specific Ni 3g sites are fully occupied by Ga atoms at *x* = 1, and further addition of Ga atoms would substitute at the remaining Ni 3g site. The Ga-Ga bonding occurs for the first time at x≧1 and the lattice expands along the *a*-axis. This scenario allows for a qualitative interpretation of the anisotropic change in the *a*-axis and *c*-axis upon substitution with Ga.

**Table 1.** Lattice parameters and unit cell volumes of CeNi_5-*x*_Ga*_x_* from this study.

Composition	*a*-axis [Å]	*c*-axis [Å]	Unit cell volume [Å^3^]
CeNi_5_	4.885	4.008	82.85
CeNi_4.5_Ga_0.5_	4.910	4.044	84.45
CeNi_4.25_Ga_0.75_	4.926	4.068	85.48
CeNi_4_Ga	4.936	4.089	86.28
CeNi_3.75_Ga_1.25_	4.991	4.095	88.34
CeNi_3.5_Ga_1.5_	5.029	4.097	89.74

### 3.2. Hydrogen absorption properties of CeNi_5-__x_Ga_x_ (x = 0, 0.5, 0.75, 1, 1.25, 1.5) alloys

**Figure 4.**
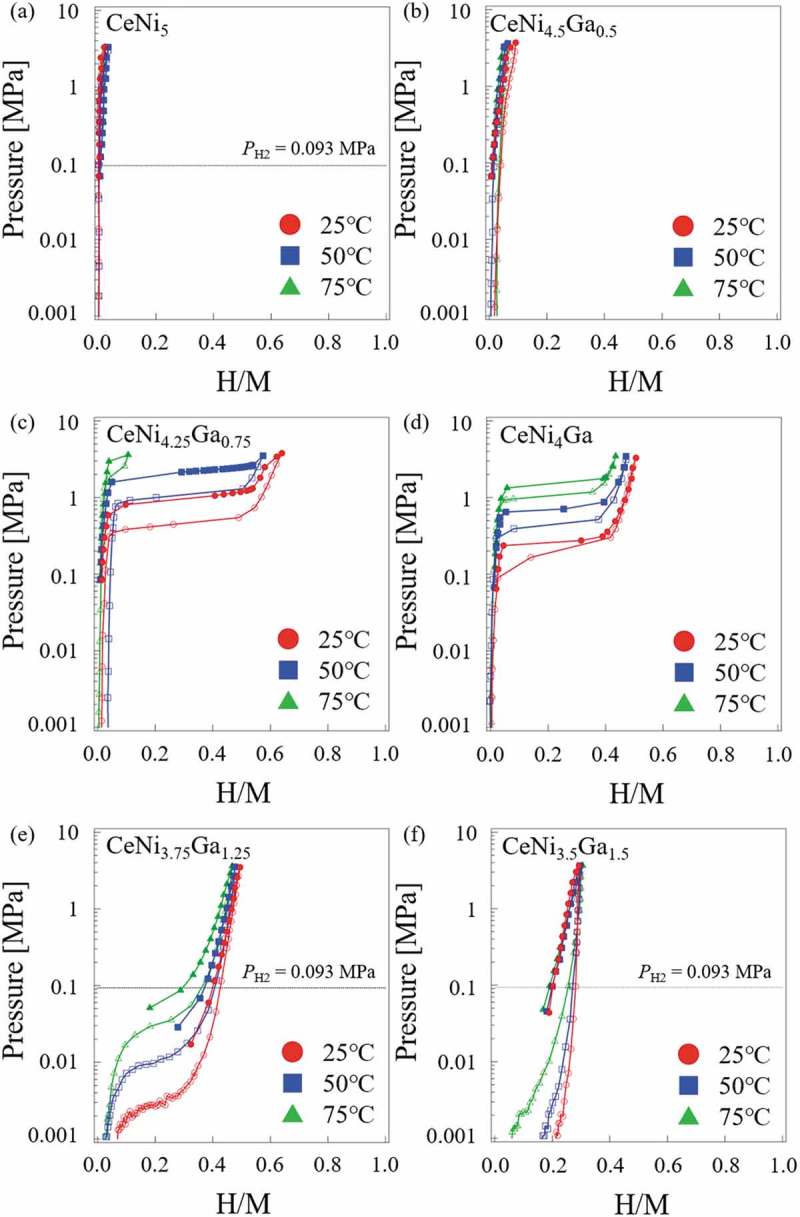
PCI measurements for CeNi_5-*x*_Ga*_x_* alloys: (a) CeNi_5_, (b) CeNi_4.5_Ga_0.5_, (c) CeNi_4.25_Ga_0.75_, (d) CeNi_4_Ga, (e) CeNi_3.75_Ga_1.25_, (f) CeNi_3.5_Ga_1.5_.

**Figure 5.**
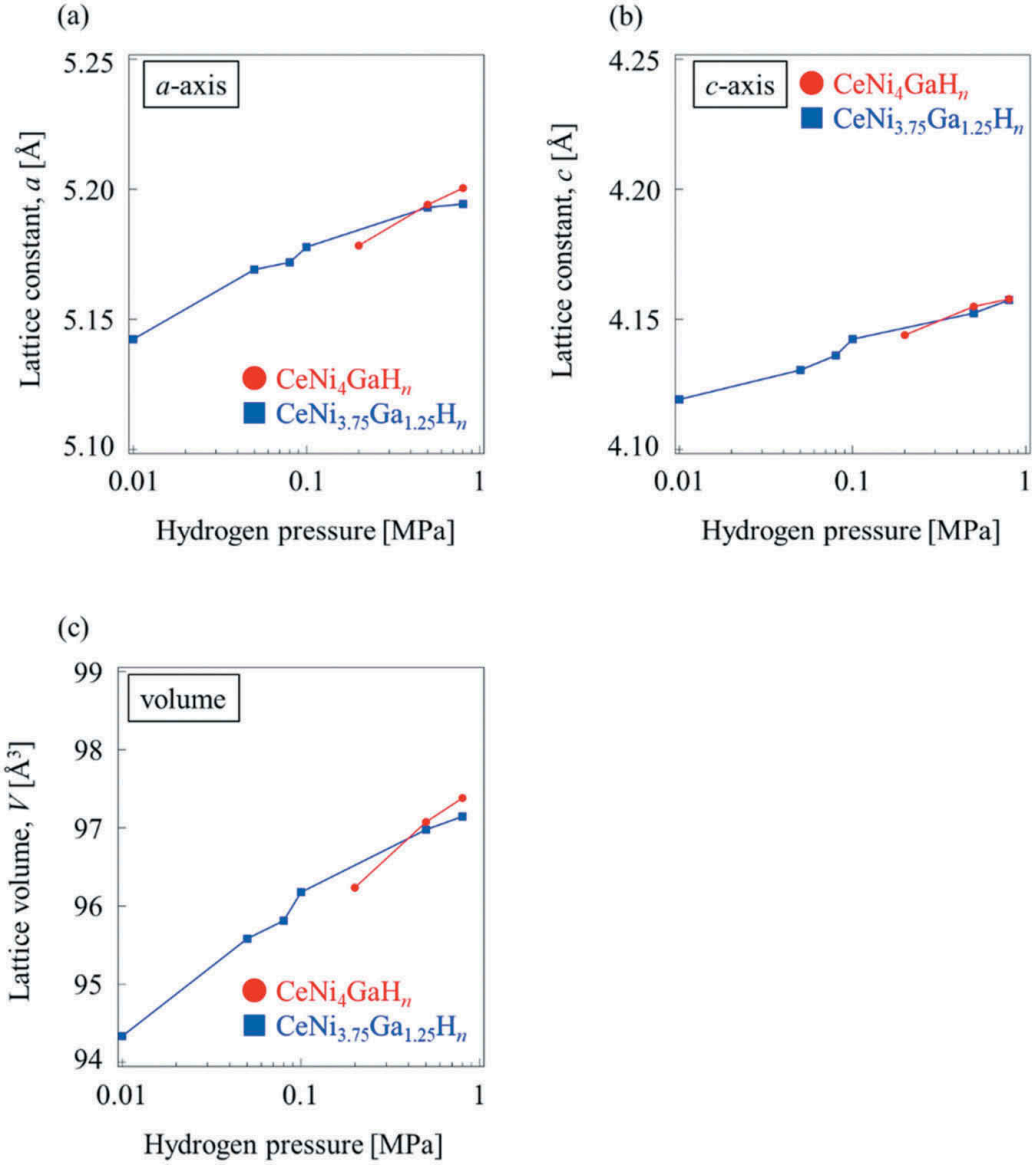
Variation in lattice parameters and unit cell volume with hydrogen pressure for CeNi_4_GaH*_n_* and CeNi_3.75_Ga_1.25_H*_n_*: (a) *a*-axis, (b) *c*-axis, (c) unit cell volume.

All CeNi_5-*x*_Ga*_x_* (*x* = 0, 0.5, 0.75, 1, 1.25, 1.5) alloys were treated with hydrogen gas under different pressures and temperatures to obtain PCI measurements. Figure 4 presents the results of PCI measurements for the CeNi_5-*x*_Ga*_x_* (*x* = 0, 0.5, 0.75, 1, 1.25, 1.5) alloys. The equilibrium plateau pressure was not observed at hydrogen pressures up to 4 MPa for CeNi_5_ and CeNi_4.5_Ga_0.5_. As shown in Figure 4, CeNi_4.25_Ga_0.75_ achieved the maximum hydrogen absorption capacity (H/M = 0.64) at 25°C under the measurement conditions. The plateau pressure also increased with measurement temperature. The variations in the lattice parameters and unit cell volume for each hydride against hydrogen pressure are also shown in Figure 5. The expansion due to pressure along the *a*-axis (Δ*a* ≦ 0.27 Å, Δ*a/a*_0_ × 100 ≦ 5.4% up to 0.8 MPa H_2_) is slightly longer than that along the *c*-axis (Δ*c* ≦ 0.07 Å, Δ*c/c*_0_ × 100 ≦ 1.7% up to 0.8 MPa H_2_). Neutron diffraction studies reports that deuterium atoms occupy the 6*m* and 12*n* site selectively in case of RENi_5_ type alloys, such as LaNi_4_AlD*_n_* [42] and LaNi_4.25_Al_0.75_D*_n_* [43]. These alloys show similar lattice expansion against hydrogen pressure, therefore hydrogen atoms in CeNi_5-*x*_Ga*_x_* are considered to occupy the 6*m* and 12*n* site.

### 3.3. Catalytic tests of CeNi_5-__x_Ga_x_ (x = 0, 1.25, 1.5) alloys

**Figure 6.**
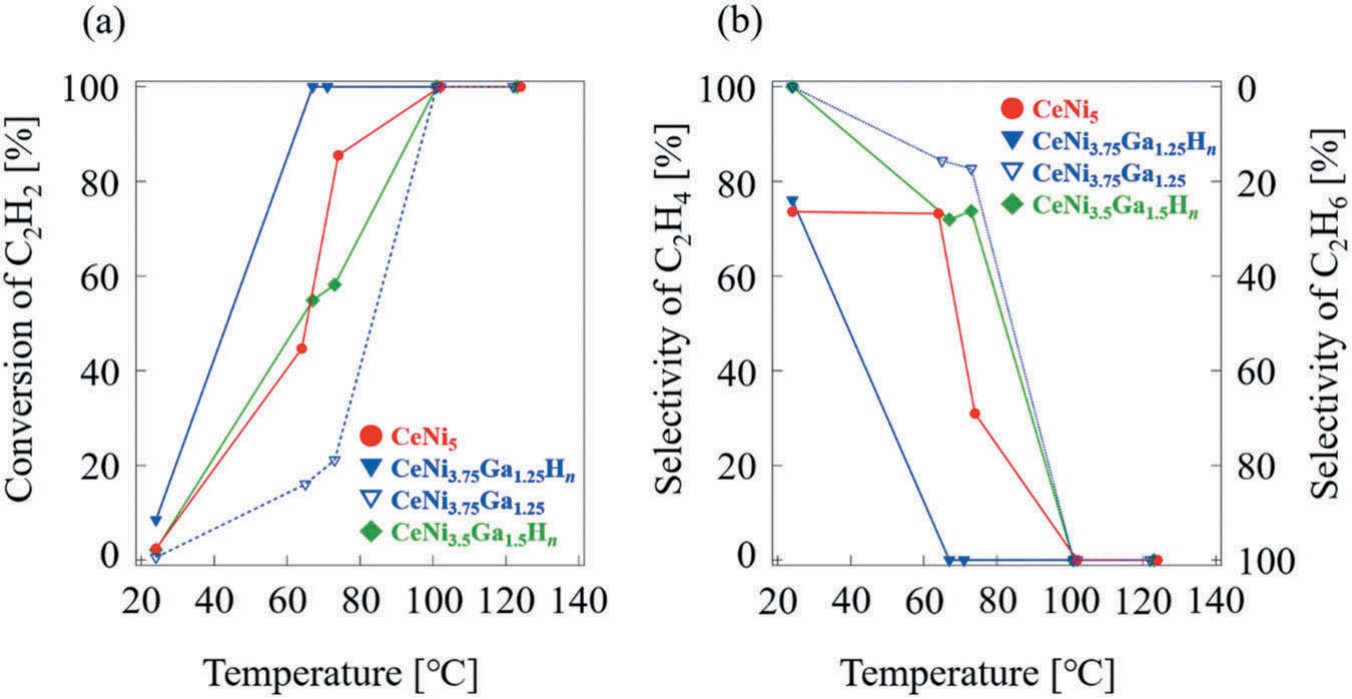
(a) Conversion and (b) selectivity of CeNi_5_, CeNi_3.75_Ga_1.25_H*_n_*, CeNi_3.5_Ga_1.5_H*_n_*, and CeNi_3.75_Ga_1.25_.

Catalytic tests for hydrogenation of C_2_H_2_ were conducted on four samples: CeNi_5_, CeNi_3.75_Ga_1.25_H*_n_*, CeNi_3.5_Ga_1.5_H*_n_*, and CeNi_3.75_Ga_1.25_. Conversion and selectivity results are shown in Figure 6. The weight of specimens and their surface areas are presented in Table 2. Selectivity corresponds to the fraction of C_2_H_4_ in the products in the gas phase. The surface area (SA) of each sample before the reaction was maintained at approximately 0.03 m^2^. The CeNi_3.75_Ga_1.25_ without exposure under high hydrogen pressure possessed lower reactivity than CeNi_5_. The CeNi_3.75_Ga_1.25_ was unable to absorb enough hydrogen because the initial activity was insufficient. The CeNi_5_ was not able to absorb hydrogen under the measurement conditions (*P*_H2_ = 0.093 MPa (Figure 4(a))). The results clearly showed that the replacement of Ga for Ni in CeNi_5-*x*_Ga*_x_* alloys decreased catalytic activity for hydrogenation. In contrast, CeNi_3.75_Ga_1.25_H*_n_* possessed greater reactivity and converted all C_2_H_2_ to C_2_H_6_ at temperatures above 50°C. The CeNi_3.75_Ga_1.25_H*_n_* alloys showed higher activity for hydrogenation of C_2_H_2_ because the absorbed hydrogen simultaneously provided dissociative atoms or subsurface hydrogen. Furthermore, the greater reactivity of CeNi_3.75_Ga_1.25_H*_n_* compared to CeNi_3.5_Ga_1.5_H*_n_* was attributed to a greater hydrogen capacity of CeNi_3.75_Ga_1.25_H*_n_*. Compared CeNi_3.75_Ga_1.25_ with CeNi_3.5_Ga_1.5_, CeNi_3.75_Ga_1.25_ had greater hydrogen capacity than did CeNi_3.5_Ga_1.5_ at *P*_H2_ = 0.093 MPa from 25 to 75°C (Figure 4(e,f), CeNi_3.75_Ga_1.25_ H/M = 0.3, CeNi_3.5_Ga_1.5_ H/M = 0.18). Alloy selectivity toward C_2_H_4_ decreased when the conversion reached 100% and the product mix was nearly all C_2_H_6_ when all of the C_2_H_2_ was converted. Similar to CeNi_5-*x*_Al*_x_*H*_n_* alloys [4], absorbed hydrogen atoms in CeNi_3.75_Ga_1.25_H*_n_* alloy also improved catalytic reactivity.

**Table 2.** Weight and BET surface area for each CeNi_5-*x*_Ga*_x_* alloy sample.

Sample	Weight [g]	a_BET_ [m^2^/g] (before reaction)	SA [m^2^] (before reaction)	a_BET_ [m^2^/g] (after reaction)	SA [m^2^] (after reaction)
CeNi_5_	0.2008	0.1459	0.029	0.1073	0.022
CeNi_3.75_Ga_1.25_H*_n_*	0.2003	0.1760	0.035	0.1720	0.034
CeNi_3.75_Ga_1.25_	0.4932	0.0715	0.035	0.1079	0.053
CeNi_3.5_Ga_1.5_H*_n_*	0.2030	0.1779	0.036	0.1281	0.026

### 3.4. Catalytic tests for Mg_2_Ni and Mg_2_NiH_4_(LT) in hydrogenation of C_2_H_2_

**Figure 7.**
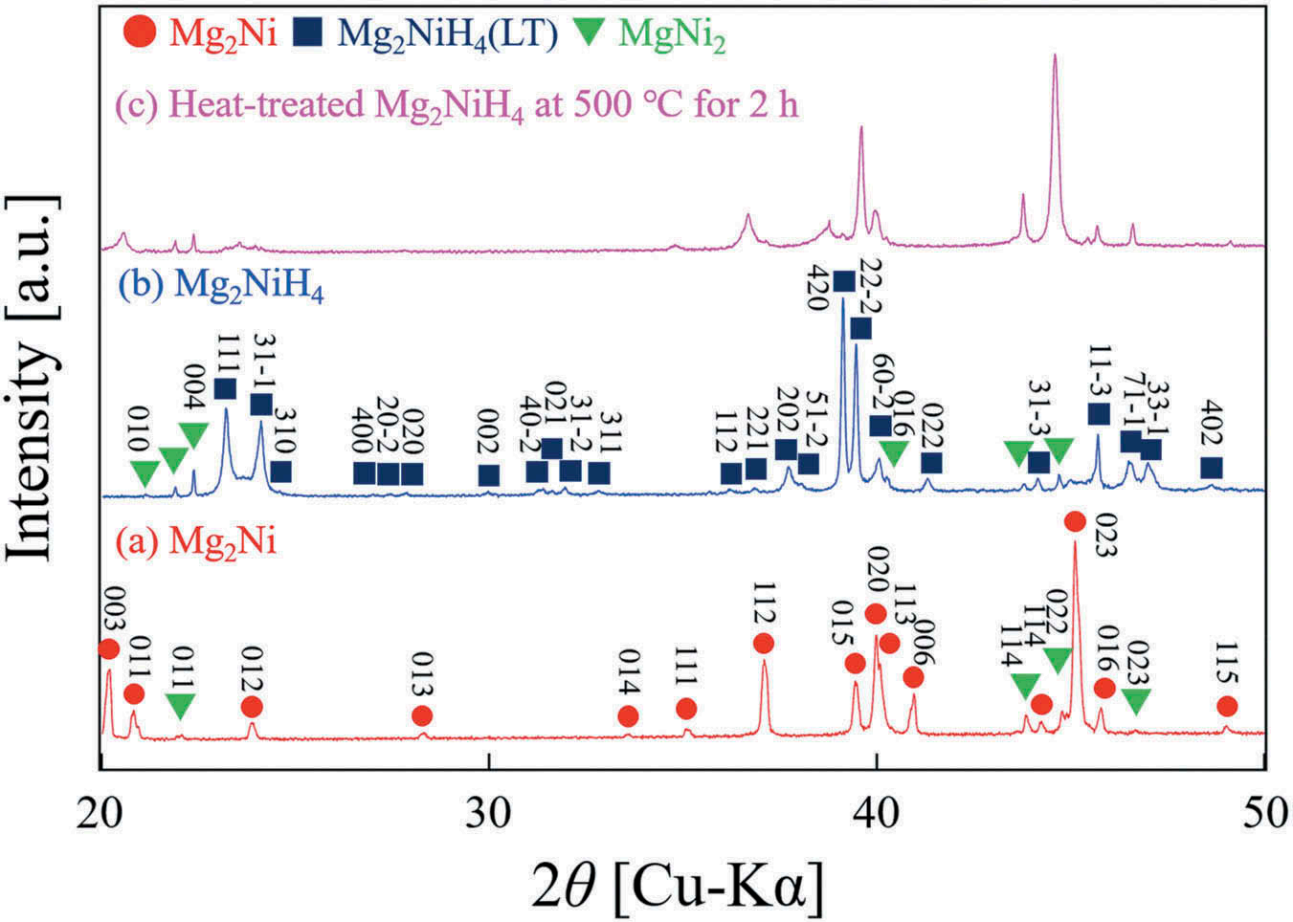
XRD patterns of Mg_2_Ni and Mg_2_NiH_4_.

**Figure 8.**
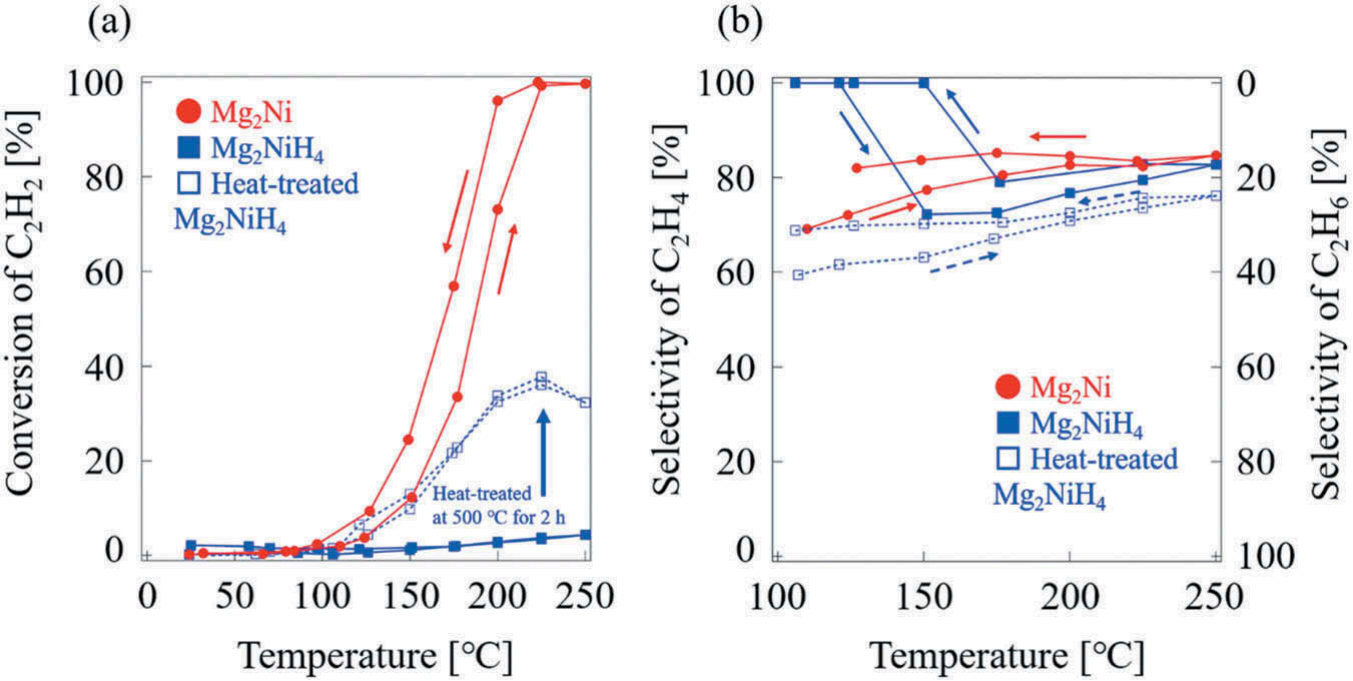
Conversion and selectivity of Mg_2_Ni and Mg_2_NiH_4_.

The powder XRD patterns for Mg_2_Ni, Mg_2_NiH_4_, and Mg_2_NiH_4_ heat-treated at 500°C for 2 h are shown in Figure 7(a–c). The Mg_2_Ni has a hexagonal structure [44], and form its hydride phase as Mg_2_NiH_4_ [45]. A phase transition in Mg_2_NiH_4_ has been observed at about 210 − 245°C [46]. The structure of the low-temperature phase, Mg_2_NiH_4_(LT), and that of the high-temperature phase, Mg_2_NiH_4_(HT), is monoclinic structure and cubic, respectively [47,48], determined by neutron scattering. A minute amount of MgNi_2_ phase was observed in Mg_2_Ni and Mg_2_NiH_4_, but its contribution could be neglected. In annealed Mg_2_NiH_4_, the peaks from the hydride phase disappeared and peaks of Mg_2_Ni shifted toward the low-angle side (Figure 7(c)), indicating that the absorbed hydrogen remained partly in matrix Mg_2_Ni phase. Figure 8 shows the results of catalytic testing and sample weight and surface areas are presented in Table 3. The Mg_2_NiH_4_ had a relatively large surface area because it had been pulverized during hydrogen absorption and desorption cycles. The surface area of Mg_2_NiH_4_ is more than twice as large as that of Mg_2_Ni prior to reaction. Surprisingly, the hydride phase of Mg_2_NiH_4_ exhibited much lower reactivity than Mg_2_Ni, as shown in Figure 8. Therefore, the formation of the hydride phase, Mg_2_NiH_4_, reduced catalytic activity in the hydrogenation of C_2_H_2_, in contrast to CeNi_5-*x*_Ga*_x_* alloys and previous reports [4,23,24,26,29,31–34,36]. After annealing, the Mg_2_NiH_4_ disappeared and Mg_2_Ni was formed, accompanied by an increase in activity in hydrogenation of C_2_H_2_. One possible reason for the reduction in reactivity is the change in the structure of Mg_2_Ni during the formation of the hydride phase.

**Table 3.** Weight and BET surface area for each Mg-Ni alloy sample.

Sample	Weight [g]	a_BET_ [m^2^/g] (before reaction)	SA [m^2^] (before reaction)	a_BET_ [m^2^/g] (after reaction)	SA [m^2^] (after reaction)
Mg_2_Ni	0.2360	0.1979	0.046	0.1516	0.036
Mg_2_NiH_4_	0.0810	1.3368	0.108	1.1266	0.091
Heat-treated Mg_2_NiH_4_	0.0822	-	-	-	-

The Mg_2_Ni selectivity toward C_2_H_4_ was greater than 80%, even at 100% conversion. Although Mg_2_Ni is an intermetallic compound composed of common metals, it possessed high activity and high C_2_H_4_ selectivity in the hydrogenation of C_2_H_2_. Selective hydrogenation of C_2_H_2_ to C_2_H_4_ is conducted industrially [49–51]. Thus, even though the test conditions are quite different than those in industrial processes, Mg_2_Ni has potential as an alternative catalyst and warrants further study.

**Figure 9.**
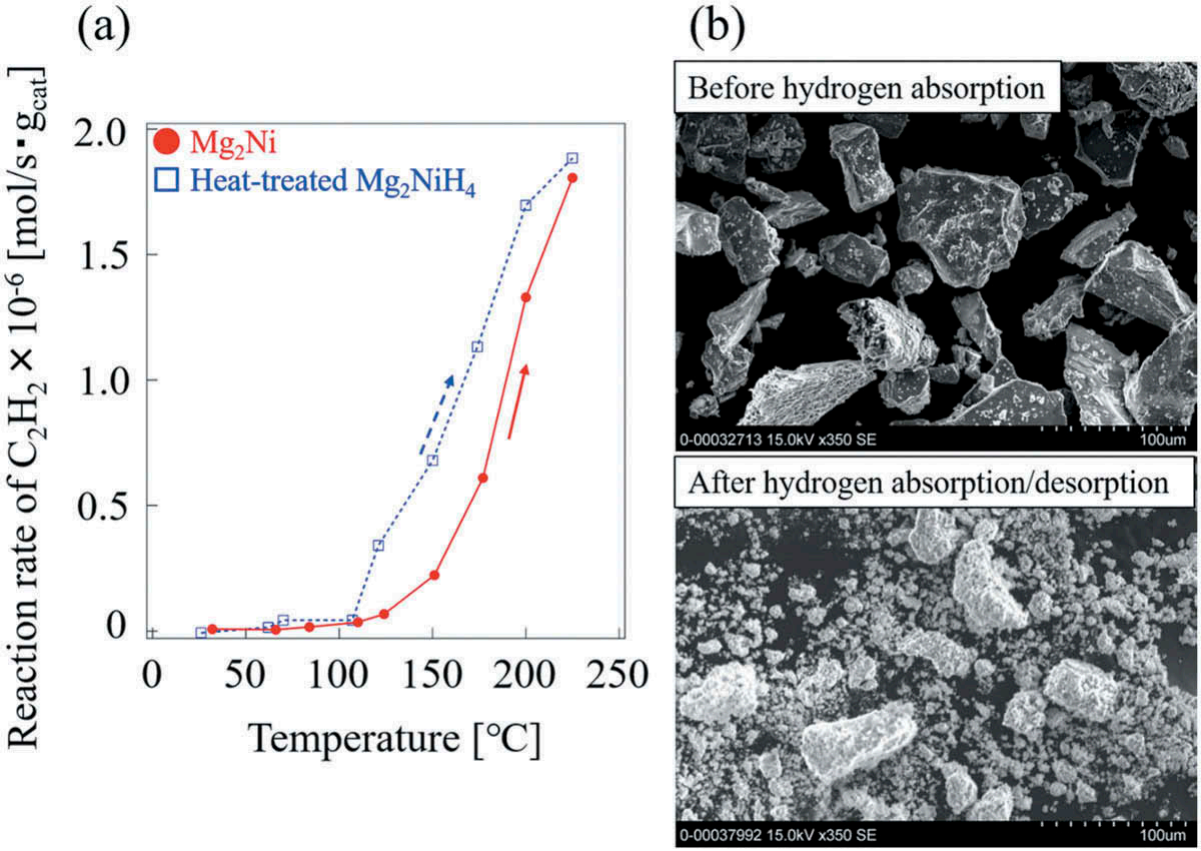
(a) Reaction rate per weight catalyst and (b) SEM micrographs of Mg_2_Ni and heat-treated Mg_2_NiH_4_ before hydrogen absorption and after five hydrogen absorption/desorption cycles.

The activities normalized for weight using Mg_2_Ni were compared with annealed Mg_2_NiH_4_ at conversion rates less than 100% (Figure 9(a)). The annealed Mg_2_NiH_4_ produced a faster reaction rate per weight of catalyst than did Mg_2_Ni, attributed to the pulverization-induced hydrogen absorption/desorption cycles that led to an increase in surface area (Figure 9(b)). The similar process, called hydrogen decomposition desorption recombination (HDDR), has been used to prepare magnet powders with high coercivity [52,53]. This can be used to obtain fine powders of alloy hydrogen-storage catalysts during the hydrogen absorption/desorption cycle. Five absorption/desorption cycles were conducted in the present study; additional cycles may produce finer samples and improve reactivity at low temperatures.

### 3.5. Transient response tests

**Figure 10.**
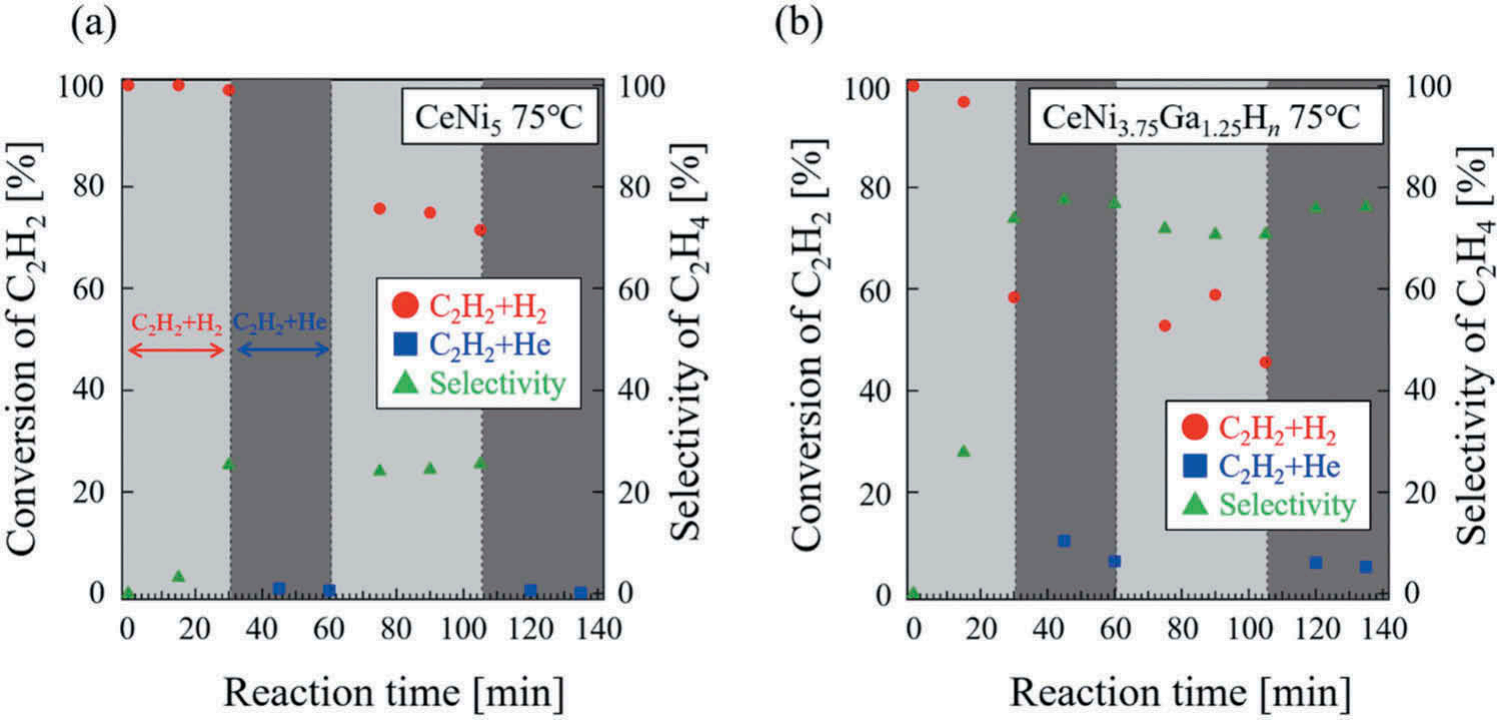
Transient response tests in CeNi_5_ and CeNi_3.75_Ga_1.25_H*_n_*.

The absorbed hydrogen may be active in the hydrogenation of C_2_H_2_ or other unsaturated hydrocarbons. If this is true, then catalytic hydrogenation can be expected to occur even in a reactant gas flow that does not contain H_2_. The latter was tested with C_2_H_2_ and He. The transient response test was performed using CeNi_5_, CeNi_3.75_Ga_1.25_H*_n_*, Mg_2_Ni, and Mg_2_NiH_4_ to understand the role of absorbed hydrogen in each catalyst. The results for CeNi_5_ and CeNi_3.75_Ga_1.25_H*_n_* are shown in Figure 10. No hydrogen absorption occurred on CeNi_5_ under the measurement conditions (*P*_H2_ = 0.093 MPa) (Figure 10(a)) and absorbed hydrogen atoms were not expected. Conversion of C_2_H_2_ over CeNi_5_ reached almost 100% under a gaseous mixture of C_2_H_2_+H_2_ (Figure 10(a)). Thus, the introduced H_2_ gas was necessary for the catalytic hydrogenation and no reaction proceeded without H_2_. However, for CeNi_3.75_Ga_1.25_H*_n_*, hydrogenation occurred under gas flow that did not contain H_2_, which indicates that hydrogenation of C_2_H_2_ originated from absorbed hydrogen in bulk CeNi_3.75_Ga_1.25_H*_n_*. The contribution of absorbed hydrogen is a relatively small and the adsorbed hydrogen is more dominant in hydrogenation over CeNi_3.75_Ga_1.25_ and CeNi_5_. This is consistent with the results shown in Figure 6, indicating that the absorbed hydrogen in CeNi_3.75_Ga_1.25_H*_n_* contributes to hydrogenation of C_2_H_2_. CeNi_3.75_Ga_1.25_H*_n_* exhibited greater activity degradation than CeNi_5_. These degradations of activity in the second cycle likely were some surface coking originated from C_2_H_2_ decomposition, which partially block adsorption sites for hydrogen. However, the reactivity over CeNi_3.75_Ga_1.25_H*_n_* under a gaseous mixture of C_2_H_2_+He is almost sustained constantly because hydrogen is driven by the large reservoir of bulk-absorbed hydrogen. As described above, the reaction with absorbed hydrogen accompanying the reaction with surface hydrogen can cause higher reactivity. However, the contribution of absorbed hydrogen is not fully clear because the atmosphere of the transient response tests (C_2_H_2_+He) is different from that of catalytic tests (C_2_H_2_+H_2_). Further studies are needed in order to reveal how much absorbed hydrogen contributes to the catalytic reaction.

**Figure 11.**
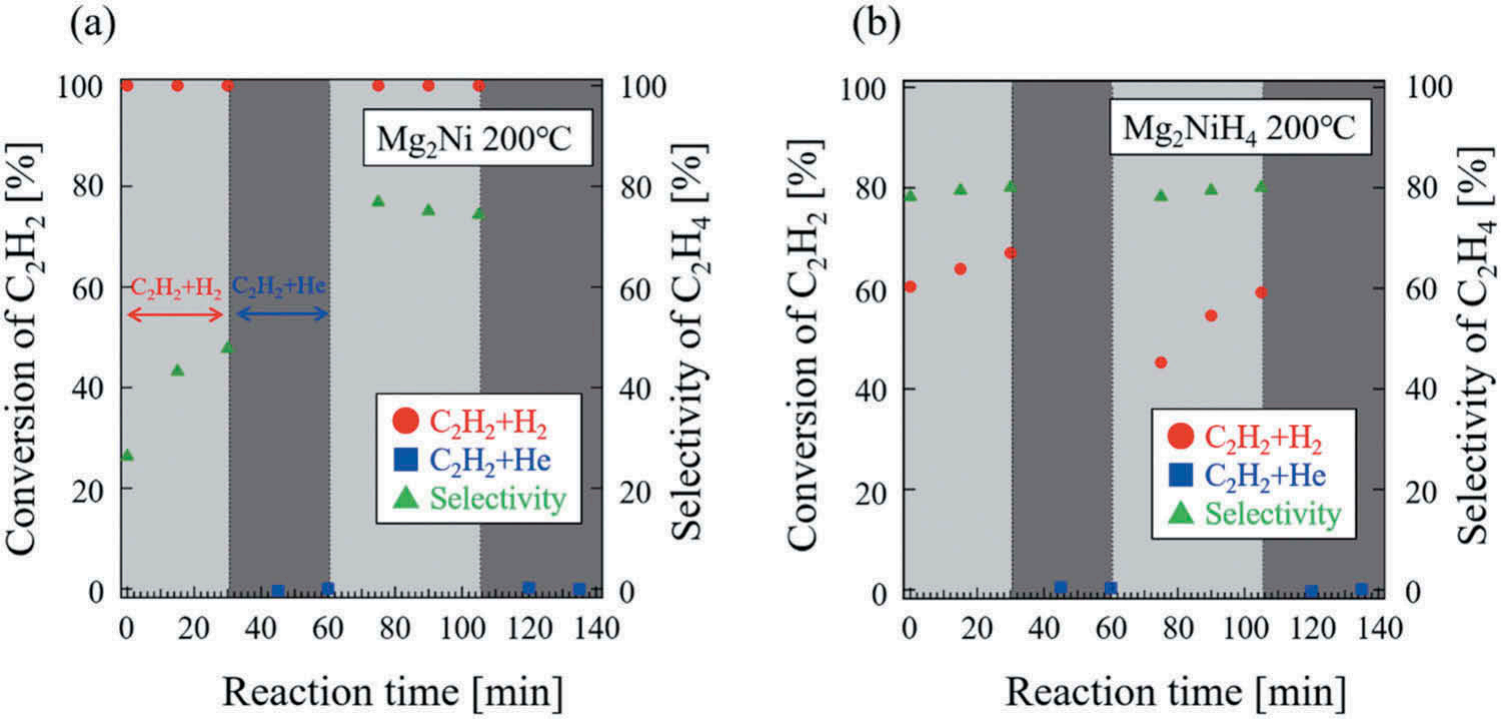
Transient response tests in Mg_2_Ni and Mg_2_NiH_4_.

The same tests were conducted over Mg_2_Ni and Mg_2_NiH_4_ (Figure 11), which revealed two interesting results. First, neither Mg_2_Ni nor the hydride phase of Mg_2_NiH_4_ possessed any reactivity under flow of a gaseous mixture of C_2_H_2_+He at any stage. Obviously, hydrogen in both Mg_2_Ni and Mg_2_NiH_4_ was not active in the hydrogenation of C_2_H_2_. Second, the hydride phase of Mg_2_NiH_4_ had significantly lower activity for hydrogenation of C_2_H_2_ than did Mg_2_Ni under a flow of gaseous C_2_H_2_+H_2_. The reason for this reactivity difference is not entirely clear. It may be due to the structural difference between Mg_2_Ni (which is hexagonal structure) and Mg_2_NiH_4_(LT) (monoclinic structure), yet a relation between the structure and the C_2_H_2_ hydrogenation reactivity would still have to be established. On the other hand, the increasing trend of the reactivity within the C_2_H_2_+H_2_ cycles (Figure 11(b)) may suggest that the Mg_2_NiH_4_ surface was initially oxidized (air-transported sample) and that it becomes gradually reduced (hence more reactive) in contact with the H_2_ gas. These results raised the question, why is absorbed hydrogen in CeNi_3.75_Ga_1.25_H*_n_*, but not in Mg_2_NiH_4_, active in hydrogenation of C_2_H_2_? A detailed mechanism has not been developed; however, one reasonable reason is the stability of absorbed hydrogen. The standard enthalpy of hydride formation derived from desorption plateau region for CeNi_3.75_Ga_1.25_H_1.5_ (H/M = 0.25) in Figure 4(e) is −39.9 ± 0.9 kJ/mol-H_2_ and that of Mg_2_NiH_4_ is −64.4 kJ/mol-H_2_ [39]. Since the stability of hydride almost depends on the enthalpy, absorbed hydrogen in Mg_2_NiH_4_ is more stable than that in CeNi_3.75_Ga_1.25_H*_n_* (the formation enthalpy of Mg_2_NiH_4_ is more negative than that of CeNi_3.75_Ga_1.25_H_1.5_) and does not seemingly react for the hydrogenation of C_2_H_2_.

## 4. Conclusions

X-ray diffraction studies of CeNi_5-*x*_Ga*_x_* (*x* = 0, 0.5, 0.75, 1, 1.25, 1.5) demonstrated substitution of Ga for Ni at the 3g site without changing the hexagonal structure (CaCu_5_-type) of P6/mmm space group. Increasing the amount of Ga substitutions caused expansion of the unit cell volume.

Catalytic and transient response tests revealed that the absorbed hydrogen in CeNi_3.75_Ga_1.25_ was consumed during hydrogenation of C_2_H_2_ and improved catalytic activity. However, the main reactant was adsorbed hydrogen and the contribution of absorbed hydrogen is a relatively small. In contrast, the formation of a hydride phase, Mg_2_NiH_4_, reduced catalytic activity and the absorbed hydrogen in Mg_2_NiH_4_ didn’t work to hydrogenation of C_2_H_2_.

Further studies are needed to understand the activity of absorbed hydrogen. Pulverization of the alloys used in the hydrogen absorption/desorption cycles is expected to increase surface area and improve reactivity at low temperature.
